# Analysis of infectious virus clones from two HIV-1 superinfection cases suggests that the primary strains have lower fitness

**DOI:** 10.1186/1742-4690-7-60

**Published:** 2010-07-20

**Authors:** Antoinette C van der Kuyl, Karolina Kozaczynska, Kevin K Ariën, Youssef Gali, Victoria R Balázs, Stefan J Dekker, Fokla Zorgdrager, Guido Vanham, Ben Berkhout, Marion Cornelissen

**Affiliations:** 1 Laboratory of Experimental Virology, Department of Medical Microbiology, Centre for Infection and Immunity Amsterdam (CINIMA), Academic Medical Centre of the University of Amsterdam, Meibergdreef 15, 1105 AZ Amsterdam, The Netherlands; 2 Department of Microbiology, Virology Unit, Institute of Tropical Medicine, Nationalestraat 155, B-2000 Antwerp, Belgium; 3 Department of Clinical Chemistry, Microbiology and Immunology, Faculty of Medicine and Health Sciences, Ghent University, De Pintelaan 185, B-9000 Gent, Belgium; 4 Faculty of Pharmaceutical, Biomedical and Veterinary Sciences, University of Antwerp and Faculty of Medical and Pharmaceutical Sciences, University of Brussels, Belgium; 5 Prosensa BV, Leiden, The Netherlands

## Abstract

**Background:**

Two HIV-1 positive patients, L and P, participating in the Amsterdam Cohort studies acquired an HIV-1 superinfection within half a year from their primary HIV-1 infection (Jurriaans *et al*., *JAIDS *2008, **47:**69-73). The aim of this study was to compare the replicative fitness of the primary and superinfecting HIV-1 strains of both patients. The use of isolate-specific primer sets indicated that the primary and secondary strains co-exist in plasma at all time points after the moment of superinfection.

**Results:**

Biological HIV-1 clones were derived from peripheral blood CD4 + T cells at different time point, and identified as the primary or secondary virus through sequence analysis. Replication competition assays were performed with selected virus pairs in PHA/IL-2 activated peripheral blood mononuclear cells (PBMC's) and analyzed with the Heteroduplex Tracking Assay (HTA) and isolate-specific PCR amplification. In both cases, we found a replicative advantage of the secondary HIV-1 strain over the primary virus. Full-length HIV-1 genomes were sequenced to find possible explanations for the difference in replication capacity. Mutations that could negatively affect viral replication were identified in the primary infecting strains. In patient L, the primary strain has two insertions in the LTR promoter, combined with a mutation in the *tat *gene that has been associated with decreased replication capacity. The primary HIV-1 strain isolated from patient P has two mutations in the LTR that have been associated with a reduced replication rate. In a luciferase assay, only the LTR from the primary virus of patient P had lower transcriptional activity compared with the superinfecting virus.

**Conclusions:**

These preliminary findings suggest the interesting scenario that superinfection occurs preferentially in patients infected with a relatively attenuated HIV-1 isolate.

## Background

Viral fitness is the parameter that is defined by the ability of an individual genotype to produce infectious progeny in a specific environment [1,2], and it can be divided into transmission fitness, replicative fitness or immune-evasion fitness. In addition to viral genetics, the host environment, i.e. type of target cells, immune response, antiretroviral drug treatment, plays an important role in viral fitness [1,2]. To measure replication fitness of HIV-1 *in vitro*, three types of assays have been developed: replication assays, single round infection assays and dual infection/competition assays [1]. The last is considered the 'gold standard' for replicative fitness determination and involves direct competition between different viral strains in cell culture infections [1,3]. For all assays, either molecular clones (virus gene of interest cloned into standard viral backbone), biological clones (single virus isolate) or a virus pool (quasi-species) can be used [1]. Competition assays have been used to determine the relative replicative fitness of viruses belonging to HIV-1 group M, HIV-1 group O and HIV-2 [4], to show that HIV-1 fitness increases during disease progression [5,6], to suggest that HIV-1 attenuates over time [7]. In contrast to the previous study, we and others have reported that viral fitness is increasing over time within the HIV-1 epidemic in The Netherlands [8,9]. This was also the case in France in 1997-2005 [10], but HIV-1 virulence was not changed over time in North America [11].

The description of HIV-1 superinfection *in vivo *is relatively new [12]. It is likely that parasites, including viruses, able to establish a productive superinfection have increased fitness over the primary infecting strain (see [13,14] and references therein). In line with this, several reports have described superinfection with a non-drug resistant HIV-1 strain in patients first infected with a drug-resistant HIV-1 strain with presumed lower fitness [15-17]. Two studies compared the relative fitness of the superinfecting strain with that of the primary strain in replication assays, but the analysis was restricted to the contribution of the *pol *gene [16,17]. In both cases no differences were observed, suggesting that fitness determining factors may be located elsewhere in the viral genome, as the superinfecting strains appeared to be more fit *in vivo*. In another superinfection case, two multidrug-resistant HIV-1 strains were involved, of which the first appeared more fit in competition assays. Not much is known about the relative fitness of the viruses in superinfection cases with HIV-1 variants lacking drug-resistance mutations. Therefore we decided to compare the replicative fitness of the primary and secondary strain in two HIV-1 superinfection cases. Biological clones were generated and *ex vivo *competition assays were performed as described earlier [5]. The *ex vivo *results were compared to the *in vivo *observations. The competition results suggest that, even though none of the strains exhibited a severe replication defect, the superinfecting virus has a higher replicative capacity than the primary strain. Analysis of the ratio of the two strains in blood plasma confirmed this finding. Full genome sequences of the viral clones were investigated to detect mutations that could explain the observed differences in replication capacity.

## Results

### Patient L

**Figure 1:**
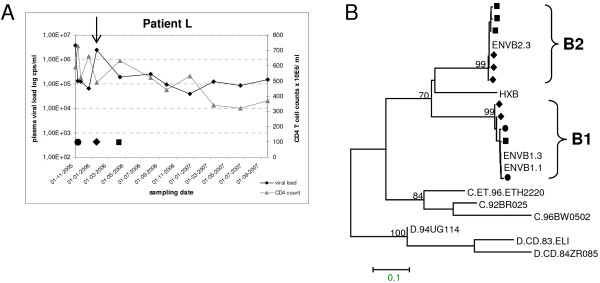
**Virological and immunological characteristics of patient L**. (A) Plasma viral load (diamonds) and CD4 + T-cell counts (triangles) of patient L from October 2005 till June 2007. An arrow indicates the probable time of HIV-1 superinfection. Biological clones were generated from PBMC samples collected in November 2005 and January 2006, respectively. (B) NJ tree constructed with representative nucleotide sequences derived from HIV-1 *env-V3 *obtained from plasma collected from patient L. Separate clusters formed by strains B1 and B2 are indicated. *Env *sequences from biological clones are indicated with clone numbers. Symbols in the tree correspond to samples from November 2005 (circles), January 2006 (diamonds) and April 2006 (squares). Reference sequences were HIV-1 strain HXB2 and subtypes C and D strains, respectively. The scale bar indicates the nucleotide distance between the sequences (as calculated with the Tamura-Nei method [59]).

Figure 1A shows the plasma viral load and CD4 + T cell count of patient L during follow up. Phylogenetic analysis of the plasma-derived HIV-1 sequences for *env-V3 *(Figure 1B) and *gag *(data not shown) were carried out on serial samples from 2005-2006. The subtype B viral sequences from 2005 cluster together and were named strain B1. A new subtype B cluster was formed by sequences from January 2006, which was named strain B2. At that time point, the new strain B2 dominated the viral population even though strain B1 could still be amplified. Three months later, in April 2006, both B1 and B2 strain sequences persisted. These observations suggest that patient L was superinfected with a second HIV-1 strain somewhere between December 2005 and January 2006, coinciding with a marked increase of the viral load (marked by a vertical arrow in Figure 1A). Similar results were obtained for the *gag *sequences (not shown).

Plasma samples from patient L were tested with strain-specific primers designed to amplify either strain B1 or B2. In December 2005 only the B1 strain was detected in both *env-V3 *and *gag *assays (not shown). At all later time-points, *gag *and *env-V3 *fragments of the B1 and B2 strain were amplified concurrently.

### Patient P

**Figure 2:**
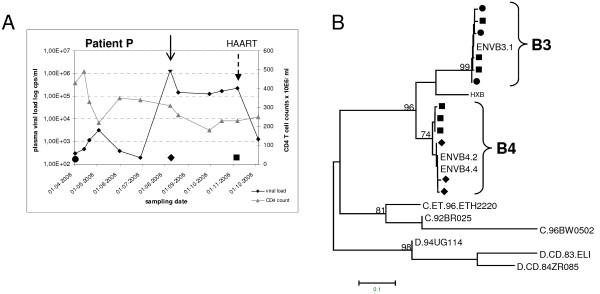
**Virological and immunological characteristics of patient P**. (A) Plasma viral load (diamonds) and CD4 + T-cell counts (triangles) of patient P from March till December 2006. An arrow indicates the probable time of HIV-1 superinfection. A second arrow indicates the start of highly active antiretroviral therapy (HAART) in November 2006. Biological clones were generated from PBMC samples collected in March 2006 (31 st of March) and August 2006, respectively. (B) NJ tree of HIV-1 *env-V3 *nucleotide fragments obtained from plasma collected from patient P. Separate clusters comprised of strains B3 and B4 are indicated. *Env *sequences from biological clones are indicated with clone numbers. Symbols in the tree correspond to samples from March 2006 (circles), August 2006 (diamonds) and November 2006 (squares). Reference sequences were from HIV-1 strain HXB2, and subtypes C and D strains, respectively. The scale bar indicates the nucleotide distance between the sequences (as calculated with the Tamura-Nei method [59]).

Figure 2A shows the plasma viral load and CD4 + T cell count of patient P during follow up. The *env-V3 *and *gag *fragments amplified from plasma samples were analysed by sequencing. Phylogenetic analysis of both gene fragments was performed on samples from March 2006, August 2006 and November 2006. Figure 2B shows a neighbour-joining tree of representative plasma-derived clones for the *env-V3 *fragment (*gag *data not shown). The sample from March 2006 showed only a single cluster - subtype B strain B3, whereas a new cluster, subtype B strain B4, was additionally present in the August 2006 sample. Three months later B3 and B4 strain sequences were amplified together. These results suggested that patient P acquired an HIV-1 superinfection between June 2006 and August 2006, concomitant with a large increase in the viral load (arrow in Figure 2B).

To estimate the ratio of strains B3 and B4 over time *in vivo*, we performed PCR on plasma samples with virus specific primers (results not shown). In a sample from March 2006 (before superinfection) only strain B3 *gag *and *env-V3 *fragments could be amplified, as expected. In plasma samples from August 2006 and November 2006 strain B4 *gag *and *env-V3 *could always be amplified, but strain B3 was probably present in lower copy numbers as it could only be amplified for *gag *(August 2006) or *env-V3 *(November 2006).

### Fitness of biological clones

Biological HIV-1 clones were generated and typed by amplifying and sequencing of *gag*, *vpr*, *env-V3*, and *nef *fragments. This confirmed their identity as the primary or secondary HIV-1 strain. Since antiretroviral drug-resistance mutations can influence HIV-1 replicative fitness we analysed the *protease-reverse transcriptase *(PR/RT) coding regions of the *pol *gene in the Stanford University HIV drug resistance database [18]. None of the clones displayed any drug resistance mutations (data not shown).

For patient L we generated approximately 200 biological clones from samples collected in November 2005 and January 2006. All clones from November 2005 appeared to contain complete strain B1 viruses (data not shown). The January 2006 sample yielded biological clones from both strain B1 and B2. We subsequently sequenced the full-length genome of a single clone (B1.1) from November 2005 and two clones (B1.3, and B2.3) from January 2006. Clones B1.1 and B1.3 consist of strain B1 sequences whereas clone B2.3 contains a complete strain B2 virus (Table 1). No B1-B2 recombinant viral clones were identified at the second time point.

**Table 1:** HIV-1 subtype B biological clones used in the ex vivo fitness experiments

Patient no.	Clone no.	Primary/superinfecting virus	Sequence analysis	Strain	Sample date
L	B1.1	primary	Complete genome	B1	Nov 2005
	B1.2	primary	Fragments only	B1	Jan 2006
	B1.3	primary	Complete genome	B1	Jan 2006
	B2.3	superinfecting	Complete genome	B2	Jan 2006
	B2.5	superinfecting	Fragments only	B2	Jan 2006

P	B3.1	primary	Complete genome	B3	March 2006
	B4.1	superinfecting	Fragments only	B4	August 2006
	B4.2	superinfecting	Complete genome	B4	August 2006
	B4.3	superinfecting	Fragments only	B4	August 2006
	B4.4	superinfecting	Complete genome	B4	August 2006

Five clones from patient L were tested for their replication capacity, alone or in competition experiments, in PHA/IL-2 activated donor PBMC's. The *ex vivo *relative fitness of HIV-1 isolates in PBMC cultures correlates with *in vivo *disease progression [5,6], making it an excellent model system with clinical relevance. The growth kinetics of individual strains indicated the absence of severe replication defects in PBMC's, although clone B1.3 replicated at a lower level compared with the other clones (result not shown).

Table 2 presents the results obtained in competitions between one of the early B1 clones (B1.1; B1.2; B1.3) and one of the late B2 clones (B2.3 and B2.5). The B2 clones outcompeted the B1 clone in all six pair-wise competitions. Clone B2.3 showed the highest relative fitness. Overall, the relative fitness of clone B2.5 was slightly lower than that of clone B2.3, but higher than that of the B1 strains. The ranking order of relative fitness is: B2.3 ≥ B2.5 > B1.1 ≥ B1.2 >> B1.3. The outcome of the competition experiments was confirmed by strain-specific PCR (data not shown).

**Table 2:** Characteristics and results of the competition experiments of selected biological clones

Clone no.	***Ex vivo *competition results **^**a**^	Replication remarks	LTR	Tat	**CCR5/CXCR4 use **^**b**^
**Patient L**					
B1.1	Against B2.3: lose	B1.1 and B1.2 replicate at similar level *ex vivo*	16 and 13 nt insertions	T23N and F32L mutations	CCR5
	Against B2.5: lose				
B1.2	Against B2.3: lose		n.d.	n.d.	CCR5
	Against B2.5: lose				
B1.3	Against B2.3: lose	B1.3 replicates at a lower level than B1.1 and B1.2 *ex vivo*.	16 and 13 nt insertions	T23N and F32L mutations	CCR5
	Against B2.5: lose				
B2.3	Against B1.1: win		Destabilizing mutation in TAR hairpin		CCR5
	Against B1.2: win				
	Against B1.3: win	B2.3 and B2.5 replicate at similar level *ex vivo*			
B2.5	Against B1.1: win		n.d.		CCR5
	Against B1.2: win				
	Against B1.3: win				

**Patient P**					
B3.1	Against B4.1: win	B3.1 replicates at very low levels *in vivo*	Destabilizing mutation in poly A hairpin	Short variant (86 aa)	CXCR4
	Against B4.2: win				
	Against B4.3: lose				
	Against B4.4: lose				
B4.1	Against B3.1: lose				CCR5
B4.2	Against B3.1: lose				CCR5
B4.3	Against B3.1: win	B4.3 and B4.4 replicate at similar level *ex vivo*			CCR5
B4.4	Against B3.1: win				CCR5

^a ^Primary clones of patient L (B1.1, B1.2 and B1.3) were competed against the superinfecting clones B2.3 and B2.5. For patient P, primary clone B3.1 was competed against all four B4 clones.

^b ^As predicted by the Geno2pheno coreceptor prediction algorithm [70]. Additionally, clone B3.1 grows in MT2 cultures, again suggestive of CXCR4 use.

From patient P, only one biological clone was generated (strain B3) from the March 2006 sample, and twenty clones were obtained from the August 2006 sample. These 20 clones were roughly analysed by amplifying and sequencing *gag*, *vpr*, *env-V3*, and *nef *genome regions, and appeared to contain complete strain B4 proviruses (data not shown). We exclusively found B4 viruses and no B3 or B3-B4 recombinant viruses amongst the biological clones from the August 2006 time-point. The only clone generated from the March 2006 sample and two clones from the August 2006 sample were completely sequenced. The single clone (B3.1) from March 2006 was confirmed to contain a strain B3 provirus and the two clones from August 2006 (B4.2 and B4.4) indeed encoded strain B4 proviruses. The fact that only a single clone was obtained from the March 2006 sample can probably be attributed to the low plasma viral load (around 10^3 ^copies/ml), which by itself could be an indication for a low replication capacity of the viral quasispecies present at that time.

A total of five biological clones from patient P were selected for the competition assays: the single clone from the first time-point and four clones from the second time-point. Individual growth kinetics of selected clones showed only modest differences between the primary and superinfecting strains, and no clone showed an obvious replication defect (not shown). Table 2 shows the results of the competition experiments where the single B3 clone, clone B3.1 was competed against four B4 clones (B4.1, B4.2, B4.3, and B4.4). The ranking order of relative fitness was: B4.4 = B4.3 > B3.1 >> B4.1 = B4.2. The outgrowth of particular virus strains was confirmed by virus strain-specific PCR (data not shown).

### Cellular gene expression profiling

**Figure 3:**
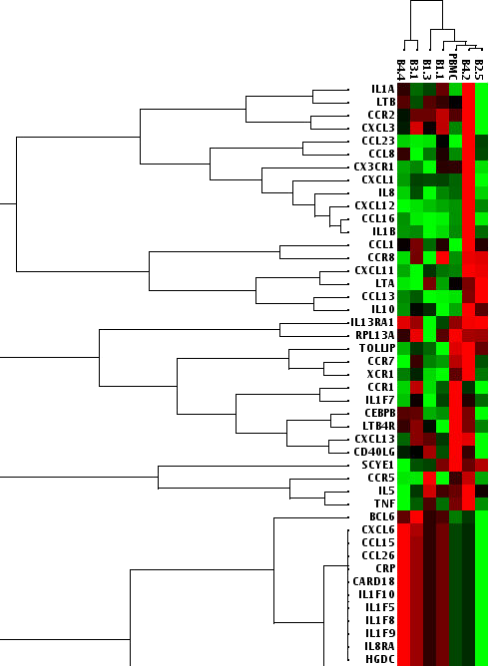
**PBMC gene expression patterns of HIV-1 biological clones**. mRNA expression levels in PBMC's infected with the HIV-1 biological clones B1.1, B1.3, B2.5 (patient L) and B3.1, B4.2, and B4.4 (patient P), as well as uninfected PBMC's were analysed with the RT^2^Profiler™ PCR Array Human Inflammatory Cytokines and Receptors (SABiosciences). Cultures were infected with HIV-1 at an MOI of 0.05. After two hours, the inoculum was removed by centrifugation. Total RNA was isolated 6 hours after infection. Experiments were performed in triplicate. Expression profiles were analysed with the GCNPro™ (Gene Network Central) software [68]. Clustering of the gene expression profiles induced by the HIV-1 clones is shown for a selection of genes from a representative experiment. Green colour indicates increased mRNA expression, red colour indicates decreased mRNA expression compared to the uninfected PBMC's.

HIV-1 is capable of modifying host cell gene expression. Micro-array data on gene modulation by HIV-1 suggest that the expression of members of multiple gene families can be changed within a few hours after virus entry (reviewed by [19]). To assess whether the ability to influence early gene expression patterns is related to viral replicative fitness, we performed a real-time PCR analysis of inflammatory cytokine and receptor mRNA's of PBMC cultures infected for 6 hours with equal TCID50 of 6 biological clones. Inflammatory cytokine genes are the most significantly upregulated genes upon HIV-1 gp120 binding to primary blood cells, and are thus a good marker of early events after viral infection. Early gene expression patterns were moderately related to the replicative fitness of the clones established earlier, whereby patterns of virus clones with lower replication capacity, e.g. B1.1 and B4.2, clustered with the patterns of uninfected control PBMC's (Figure 3). The patterns induced by potently replicating viruses, B3.1 and B4.4, clustered together and away from uninfected PBMC's (Figure 3). Clone B1.3, demonstrating an intermediate replication capacity, indeed clustered in the gene expression assay between the low and high replicating clones (Figure 3). The only exception was clone B2.5 that showed a good replicative fitness, yet yielded an early gene expression pattern that was more similar to uninfected cells. There clearly is a difference between early events (receptor binding and internalization) and virus replication, suggesting that clone B2.5 is somewhat delayed early in infection, but then has an above average replicative capacity. Although expression levels varied at the single gene level, a few mRNA's, e.g. those for CCL4, CCL5, CCL18, and IL9, were upregulated in all infected cultures compared to uninfected PBMC's.

### HIV-1 sequence analysis

**Figure 4:**
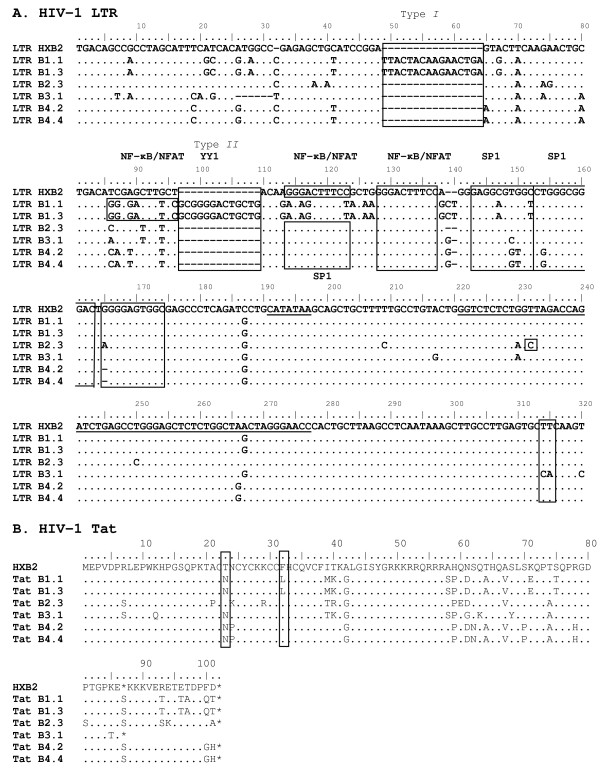
**HIV-1 LTR and Tat sequences**. (A) Nucleotide sequences of the LTR region from clones B1.1, B1.3, B2.3 (primary and superinfecting strain from patient L, respectively), and clones B3.1, B4.2, and B4.4 (primary and superinfecting strain from patient P, respectively). Sequences were aligned using the HXB2 sequence (GenBank acc. no. K03455) as reference. Binding sites for transcription factors and the two insertions found in clone B1.1 (*type I *and *type II*) have been boxed. A NF-κB/NFAT binding site immediately followed by an YY1 binding site found only in clone B1.1, are indicated. The TATA-box and the TAR region (nt 504-555) have been underlined. A destabilizing T→C mutation in the TAR hairpin region in clone B2.3 is boxed. The polyA hairpin (nt 556-602) is shown in bold, a box indicates the destabilizing TT→CA mutation in clone B3.1. (B) Translated amino acid sequences are shown for HIV-1 Tat. Sequences have been aligned with the HXB2 sequence. Clone numbers are indicated. Strains B1 and B2 are the first and superinfecting virus from patient L, respectively. Strains B3 and B4 are the first and superinfecting virus from patient P, respectively. The Tat T23 N and F32 L mutations in strain B1 associated with increased and decreased Tat activity have been boxed.

Complete genomes of the two virus strains from each patient were sequenced to identify mutations. The most interesting findings are discussed. For patient L, the LTR promoter sequences revealed two insertions of 16 and 13 nucleotides (nt), respectively, in the low replicating B1 clone compared with B2 viruses and with the HXB2 reference sequence (Figure 4A). Moderate insertions in the LTR are not uncommon in HIV-1 and have been associated with disease attenuation [20]. The insertions in the B1 LTR occur at the type I and type II insertion sites described by Koken *et al*. [3], but are dissimilar in nucleotide sequence. The LTR insertions do not affect the *nef *open reading frame. Interestingly, the second insertion together with upstream sequences creates a novel NF-κB/NFAT binding site whereas the downstream common NF-κB/NFAT binding site is hypermutated at 4 nucleotides (Figure 4A). The type I insertion in the B1 LTR is very similar to that described for a virus with decreased transcriptional activity that was isolated from a long-term non-progressing patient (no. 4) [20]. Seven additional B1 biological clones contained identical LTR sequences, indicating that the insertions in this region are not unique to clones B1.1 and B1.3 (result not shown). Clone B2.3 contains a T→C mutation in the TAR region of the LTR that could destabilize the hairpin secondary structure (Figure 4A). HIV-1 Tat protein activates transcription by binding to the TAR hairpin in the LTR, thereby acting as a potent activator of viral gene expression. Mutational analysis of four highly conserved aromatic amino acid residues within the Tat activation domain showed that the F32 L mutation greatly reduced Tat activity and virus replication [21]. Interestingly, this F32 L mutation is present in 15% of the subtype B *tat *sequences from 2008 [22]. The same mutation is also present in the B1 clones of patient L (Figure 4B), suggesting that the B1 strain encodes a Tat protein with decreased transcription activation capacity. However, the T23 N substitution in strain B1 Tat could possibly compensate for the F32 L mutation [23].

**Figure 5:**
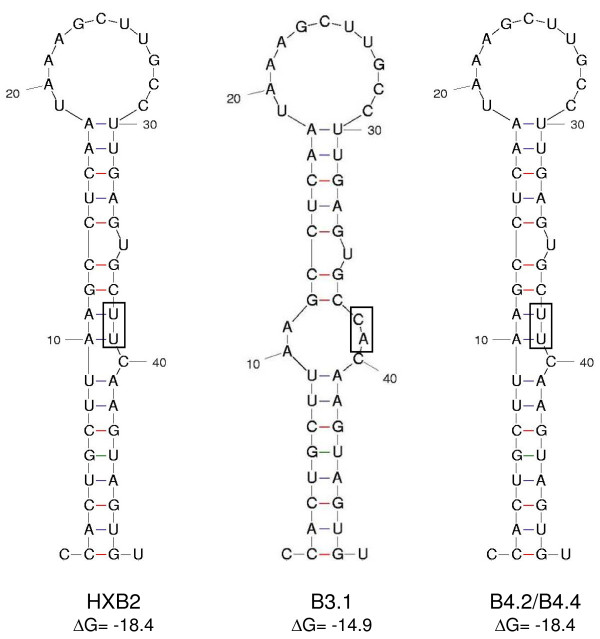
**Structure of the LTR polyA hairpin**. Predicted structure of the LTR polyA hairpin region of the HIV-1 reference strain HXB2 and clones B3.1, B4.2 and B4.4. The free energies of the stem-loop structures were calculated with the Zuker algorithm as available at the mfold webserver for nucleic acid folding and hybridization prediction [69], the ΔG values are presented in kilocalories per mole. A box indicates the UU→CA change in clone B3.1.

**Figure 6:**
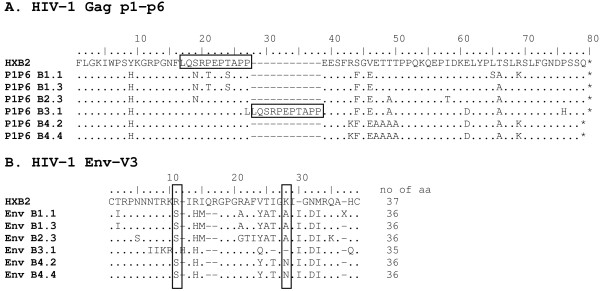
**HIV-1 Gag p1-p6 and Env-V3 sequences**. Translated amino acid sequences are shown for HIV-1 Gag p1-p6 region (panel A), and env-V3 (panel B). Sequences were aligned with the HXB2 reference sequence. Clone numbers are indicated. Strains B1 and B2 are the first and superinfecting virus from patient L, respectively. Strains B3 and B4 are the first and superinfecting virus from patient P, respectively. The 11 aa PTAPP repeat in clone B3.1 in Gag-p6 has been boxed. The 11^th ^and 25^th ^amino acid residues in Env-V3, associated with CXCR4 coreceptor use when positively charged, are indicated.

For patient P, the LTR promoter sequence of the first infecting virus, strain B3, carried a characteristic TT→CA mutation in the poly A hairpin region (Figure 4A). Such a mutation destabilizes the structure of this hairpin (Figure 5), which may trigger premature polyadenylation in the 5' LTR thus reducing viral gene expression and replication [24,25]. Analysis of the plasma viral quasispecies at the first time point (when only the B3 strain is present) revealed that all 16 HIV-1 LTR clones analysed contained the TT→CA substitution in the LTR (not shown). The Tat protein encoded by the B3.1 virus clone has 86 amino acid residues, while the B4 clones encode a Tat protein of 101 amino acid residues (Figure 4B). As such a short *tat *gene was initially observed in laboratory strains, it was suggested that a shorter Tat protein was sufficient only for *ex vivo *propagation of the virus (reviewed by [26]). A premature stopcodon at position 86 of the *tat *gene occurs occasionally in all subtypes, and regularly in almost all subtype D isolates [22]. In addition, clone B3.1 has an 11-codon repeat of the 'PTAP' motif at the beginning of the *gag-p6 *protein reading frame that is not present in the B4 strain (Figure 6A). A sequence repeat of 3-9 amino acid residues at this location has been associated with low CD4 + T cell counts, drug resistance and poor prognosis [27-29]. Interestingly, gag-p6 PTAP repeats have linked to the presence of positively charged amino acid residues at certain positions in the env-V3 loop that determine co-receptor usage [27]. The 11^th ^position in the V3 loop of the B3.1 clone encodes the positively charged R residue, suggesting CXCR4-usage [30-32], but the 25^th ^position could not be clearly assigned to a charged amino acid [27,31,32] (Figure 6B). Indeed, clone B3.1 infected MT-2 cell cultures with induction of syncytia, indicative of CXCR4 use (result not shown). We were, however, unable to infect U87.CD4 cells expressing either CXCR4 or CCR5 [33] with this clone. The V3-loop of clone B3.1 has remarkable similarity to that of subtype D virus UG21 that can use the APJ and CCR9 receptor in addition to CXCR4 [34], suggesting it could be different from common CXCR4 using strains, and possibly have less affinity for U87.CXCR4 cells. The secondary virus strain B4 of patient P was predicted to use the CCR5 coreceptor, as were both primary and secondary strains of patient L, but this was not tested in culture. Analysis of viral RNA present in blood plasma at the first time point confirmed that the *env-V3 *sequence of clone B3.1 is present in all viral genomes analysed (result not shown). No apparent escape mutations were seen in Gag epitopes defined by the patients HLA type, suggestive of low CTL pressure.

Another intriguing finding is the difference in replication capacity of clones B1.1 and B1.3, where the latter clone exhibits a substantial *ex vivo *replication disadvantage in competition experiments. Yet relatively little sequence variation was found that could account for this. A single amino acid difference was noted in the Vpu and Rev proteins, as well as 8 amino acid differences in Env (3 in gp120, 5 in gp41). The genetic difference between clones B4.2 and B4.4, of which the former clone has a replicative disadvantage, was also modest. In addition to a single amino acid difference in Vif and one in Vpu, two amino acid changes were found in the *env *gene, one in the signal peptide and one in the *env-V5 *domain, respectively. Also, an extra glycosylation site was present in the env-V4 region of clone B4.4. The HIV quasispecies in a host consist of many closely related variants, and (modest) differences in replication capacity are to be expected. Replication curves of single clones, e.g. B4.2 and B4.4, did not show significant differences in replication when cultured alone (not shown). However, competition experiments can expose and enlarge relatively small differences in replication capacity [3]. Therefore, that two out of four B4 clones lost in competition experiments from the same clone, while two other B4 clones won the competition does not represent evidence that the former B4 clones are largely deficient in replicative capacity.

### LTR promoter activity

**Figure 7:**
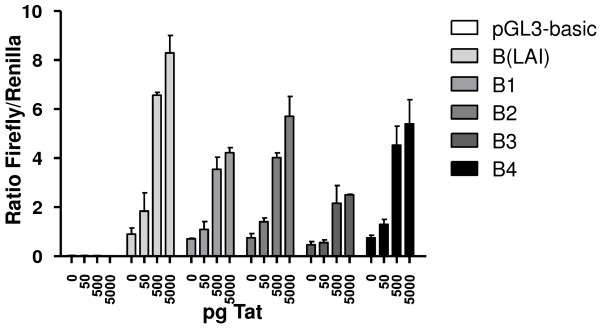
**Transcriptional activity of the LTR promoter sequences**. Transcriptional activity of the HIV-1 LTR promoter sequences from HIV strains B(LAI), B1, B2, B3, and B4, compared with the empty vector (pGL3-basic) in a dual firefly/renilla luciferase assay. LTR fragments cloned from clones B1.1/B1.3 and B4.2/B4.4 are identical in sequence, so only strain names are indicated. Transcriptional activity of the luciferase gene was tested in the presence of increasing concentrations of Tat. The value is the average of three independent measurements; standard deviations are indicated.

The promoter activity of the LTR region of the primary and superinfecting HIV strains was analysed by cloning a fragment corresponding to nt 2-536 of the HXB2 genome before the luciferase gene and subsequently measuring luciferase activity in the presence of increasing amounts of Tat (Figure 7). There is no obvious difference between the LTRs from the B1 and B2 strain from patient L in the human embryonic kidney cell line used, despite the occurrence of insertions in the B1 LTR. However, the LTR from the primary virus B3 from patient P has a lower promoter activity than the LTR from the superinfecting virus B4 in these cells, despite the absence of noticeable sequence variation. Using different cell types and/or activating the promoters with homologous Tat protein instead of HIV(LAI) Tat could influence the results, as promoter activity has not only been shown to be cell-type specific, but there might also be co-evolution between the LTR and *tat *gene of a particular HIV strain. For example, it would be very informative to analyse LTR activity in PMA and/or ionomycin stimulated cells, preferentially in a T-cell line, to determine the true effect of NF-κB and NFAT upon transcription.

## Discussion

In the present study the relative fitness of viral strains involved in two HIV-1 superinfection cases was analysed. Patients L and P were identified to have experienced an HIV-1 superinfection within half a year from the seroconversion date by a sudden unexpected rise in the plasma viral load [35]. Both virus strains in the two patients are supposedly "wild-type" viruses, meaning that no drug-resistance mutations in *pol *or deletions or premature stopcodons in the *nef *gene were found. Therefore, these HIV-1 strains are well suited to test the hypothesis that productive superinfection requires a second virus with a higher relative fitness than the primary infecting strain [13,14]. The presence of an initial virus strain with drug-resistance mutations, likely causing a replication disadvantage, has been reported repeatedly [15-17].

To estimate the relative fitness of the virus variants involved in two HIV-1 superinfections, replication competent viruses were obtained by biological cloning. An initial genome analysis was performed by PCR amplification of gene fragments and subsequent sequence analysis. For patient L, around 200 clones were obtained in line with the relatively high plasma viral load at the time points sampled (> 10^5 ^copies/ml). Strain B1 clones were isolated before and after the superinfection moment, strain B2 only after superinfection. Both the *in vitro *competition experiments with multiple pairs of B1 and B2 clones and the ratio of the two strains in blood plasma samples indicated that the second strain B2 is the better replicating strain, in line with the hypothesis that a more virulent strain can infect a host that is already infected with a less virulent strain [13,14]. For patient P the situation turned out to be more complex. A major restriction is that only a single clone of the initial B3 virus was obtained. This is probably due to the extremely low viral load. In fact, the viral load remains low for many months before superinfection occurs. In blood plasma, *gag *and *env-V3 *fragments from the B3 strain could not be amplified from all samples, confirming low copy numbers of this strain. In contrast, strain B4 sequences were abundantly present in plasma samples taken after the superinfection moment. This observation, together with the sustained increased plasma viral load, suggests a significantly higher level of replication of the second strain. In *ex vivo *experiments, the single and possibly unusual B3 clone was able to outgrow two B4 clones in the replication assays, although it appeared less fit than two other B4 clones. Probably, the single B3 clone isolated is one of the better replicating variants of the quasispecies, and thus not fully representative of the B3 quasispecies of patient P *in vivo*. In a luciferase assay using human embryonic kidney cells, the B3.1 LTR was less active as a promoter than an LTR from the B4 strain, which could suggest that this could also be the case in the various cell types infected *in vivo*. Alternatively, strain B3 may have an average replication capacity, but is severely suppressed *in vivo *by the immune system resulting in the low plasma viral RNA levels observed. A second strain could experience less immune pressure such that it can replicate to higher levels [17]. However, no primary or secondary clone possessed escape mutations in the major gag or nef epitopes targeted by the HLA-A25, -B18 or -B44 alleles carried by the patient [36-40], thus suggesting ineffective cytotoxic T cell responses (result not shown). Both the *env-V3 *sequence and culture experiments using MT2 cells suggested that clone B3.1 uses the CXCR4 coreceptor, although patient P does not carry CCR5-Δ32 deletion alleles (not shown). Primary infections with CXCR4-using viruses are not unusual, as thought earlier, although they are usually negatively selected during primary infection. Over 15% of patients with a primary HIV infection in two European cohorts were infected with CXCR4-using strains [41,42]. CXCR4-using viruses are not necessarily more fit than CCR5-using viruses. Competition experiments with biological clones showed that the average fitness of CXCR4- and CCR5-using viruses is similar [43]. In conclusion, the combined data suggest that overall the superinfecting virus in patient P is also a better replicating strain than the primary virus.

HIV-1 superinfection has been associated with disease progression, as exemplified by a permanent rise in the plasma viral load and an accelerated decrease in CD4 + T cell numbers (reviewed in [44]). Mathematical modelling suggests that, except for the direct negative effect of accelerated disease progression, co- and super- infections can also have an impact on the virus as a species in the epidemic, triggering an increased replication capacity and possibly virulence of the pathogen [14]. *In vitro *experiments with vesicular stomatitis virus (VSV) show that the progeny of co- and super- infections, have a higher fitness than that of single infections as the dual infections allow for faster adaptation by to environmental changes [45]. Low viral fitness, measured as replicative capacity, is associated with lower virulence, e.g. in *nef*-deleted HIV-1 or drug-resistant HIV-1 variants [46,47]. Studies on HIV-1 fitness and evolution have been contradictory. A initial study suggested attenuation of HIV-1 over time in Belgium [7], but other studies reported increasing fitness of HIV-1 in The Netherlands in the period 1986-2003 [8,9] and in France in 1997-2005 [10]. A fourth study indicated that HIV-1 virulence is not changing over time in North America [11]. As HIV-1 co- and super- infections are much more prevalent in Africa (reviewed in [44]), it will be of interest to study the evolution of viral fitness in this setting.

## Conclusions

The results obtained from two HIV-1 superinfection cases suggest that an HIV-1 re-infection that gives rise to a systemic superinfection is facilitated by a primary infection with a less fit strain that has a lower replication capacity than the superinfecting strain. It remains important to examine the replication capacity of viruses from other patients with an HIV-1 superinfection to see if the suggestion of a better replicating second virus can be confirmed.

## Materials and methods

### Patients

Two HIV-1 positive patients, L and P, were found to have an HIV-1 superinfection in an earlier study analysing sudden plasma viral load rises in patients followed at the Academic Medical Center in Amsterdam, The Netherlands [35]. Both individuals initially presented with primary HIV-1 subtype B infections; patient L with Fiebig stage II (vRNA^+ ^) and patient P with Fiebig stage V (Western blot^+ ^p31^-^) [48]. Both experienced an HIV-1 superinfection with another subtype B strain within half a year from their first presentation. The patients participated in the Amsterdam Cohort Studies (ACS) on HIV infection and AIDS among homosexual men. Written informed consent was obtained. HLA-typing of ACS patients with a primary HIV-1 infection is routinely performed at Sanquin Diagnostiek (Amsterdam, The Netherlands). Patient L: HLA class I: A2, A24(9), B27, B60(40), Cw 1 and 7; HLA class II: DR1, DR12(5), DR52, DQ5(1), DQ7(3). Patient P: HLA class I: A2, A25(10), B44(12), B18, Cw5; HLA class II: DR15(2), DR12(5), DR51, DR52, DQ1 and DQ3. HIV-1 blood plasma viral load measurements were done at the Laboratory of Clinical Virology at the AMC (Amsterdam, The Netherlands) with the Versant HIV-1 RNA 3.0 assay (Bayer Diagnostics Division Tarrytown, N.Y.).

### Cloning and sequencing of molecular clones from plasma

RNA was isolated from plasma samples from both patients with a method using silica and guanidium thiocyanate [49]. HIV-1 *env-V3 *and *gag *fragments were reverse transcribed, amplified, cloned with the TOPO TA cloning kit (Invitrogen, Carlsbad, Calif.), and sequenced with the BigDye Terminator cycle sequencing kit (Applied Biosystems, Foster City, Calif.) as described [50,51]. Electrophoresis and data collection were performed on an ABI PRISM 3100 genetic analyser (also from Applied Biosystems). At least 16 clones were analysed for each patient per time point and per strain.

### Generation of biological clones

Freshly phytohemagglutinin (bioTRADING Benelux, Mijdrecht, The Netherlands), glutamax and interleukin-2 (Proleukin, Chiron, Emeryville, Calif.) stimulated peripheral blood mononuclear cells (PBMC's), obtained from four healthy (HIV-1 negative) human donors, were combined and cultured in RPMI 1640 medium (Invitrogen Corporation, Carlsbad, Calif.) supplemented with antibiotics, L-glutamine and 15% heat-inactivated foetal calf serum for 3 days. CD8 + T cells were depleted after 2 days using the Dynabeads M-450 CD8 kit (Invitrogen Corporation, Carlsbad, Calif.). Different concentrations of PBMC's from the HIV-1 infected patient (10^4^, 2.5 × 10^4^, 4 × 10^4 ^6 × 10^4 ^cells/well) were cocultivated with 1 × 10^6 ^CD4 + T cells in the same medium in 96-wells plates for 21 and 28 days, respectively. Each 7 days culture supernatants were tested for the presence of p24 with an in-house antigen capture enzyme-linked immunosorbent assay (ELISA). At the same time, to propagate the culture, one-third of the cell culture was transferred to new 96-well plates and fresh PHA, IL-2 stimulated CD4 + T cells were added. Viruses were considered to be clonal if less than one-third of the microcultures became positive at a given cell number (Poisson distribution). HIV-1 clones were expanded by culturing and harvested after 7 days [52]. PBMC's and supernatant were cryopreserved at -150°C [53].

### Analysis of *gag*, *env*, *vpr *and *nef*

To characterize the biological clones, a 804 nucleotide fragment of the *gag *gene, encompassing the entire *p17 *gene and the 5' part of the *p24 *gene, and a 264 nucleotide HIV-1 *V3 *fragment of the *env *gene were amplified by PCR as previously described [50,51,54]. The complete *vpr *gene of the biological clones was amplified and sequenced [55]. The *nef *gene was amplified using 5' End-env-s primer (5'TAG AAG AAT AAG ACA GGG CTT GG3') and R-LTR3'FM (5'AGA CCC AGT ACA GGC AAA AAG CAG CTG CTT ATA3') using Amplitaq (Perkin Elmer 5 units/μl) in a final concentration of 1.8 mM MgCl_2_. Amplification was done for 40 cycles, with each cycle involving three steps: 1 min at 95°C, 1 min at 55°C, and 2 min at 72°C, plus a final extension of 10 min at 72°C.

### Full genome sequencing

The complete genomes of three biological clones for each patient were sequenced (for patient L clones: B1.1, B1.3, and B2.3, for patient P clones: B3.1, B4.2 and B4.4; Table 1). Overlapping fragments of about 600-1000 bp in length, spanning the entire genome, were amplified with different primer sets and sequenced. Sequences were compiled with CodonCode Aligner version 2.0.3 [56]. Differences between the primary and superinfecting strain, represented by clones B1.1 and B2.3 from patient L and clones B3.1 and B4.2 from patient P is discussed in the Results section. Differences observed between clones of the same strain (B1.1 vs. B1.3 and B4.2 vs. B4.4) are discussed in the Results section as well.

### Phylogenetic analysis

Sequences were aligned with the CLUSTAL W sequence alignment tool implemented in BioEdit Sequence Alignment Editor Version 7.0.9 [57]. Reference sequences were obtained from the Los Alamos HIV sequence database [22]. The alignments were manually adjusted to preserve in-frame insertions and deletions. Phylogenetic analyses were performed with the MEGA4 software package distributed by Sudhir Kumar, Arizona State University, Tempe [58]. Distances were estimated with the Tamura-Nei method [59], using the gamma model to correct for multiple hits and to account for excess transitions, unequal nucleotide frequencies, and variation of substitution rate among different sites. For the shape parameter alpha, which describes the variation across sites by a gamma distribution, we used α = 0.38 for *env-V3 *and α = 0.25 for *gag *[60]. Phylogenetic trees were generated with the neighbour-joining method and bootstrap resampling with 1000 replicates. Phylogenetic analyses of *vpr *and *vpu *genes were performed in a similar fashion.

Transcription factor binding sites were identified in the long terminal repeat (LTR) sequences with TFSEARCH [61] and Alibaba 2.1 [62].

### Virus replication assays

Replication kinetics of biological clones was measured by infecting pooled PHA/IL-2 stimulated PBMC's from at least four healthy donors. Similar amounts of p24 were used for each viral strain. After 4 hours of infection, the inoculum was removed by centrifugation. PBMC's were resuspended in complete medium and cultured for 16 days. Virus production was analysed with an in-house p24 antigen capture enzyme-linked immunosorbent assay (ELISA) at days 0, 2, 6, 9, 13 and 16.

### Growth competition assays

Different HIV-1 biological clones generated as described above were selected based on their *env-V3 *sequence and the replicative fitness was assessed in growth competition assays. The infectivity of each virus was determined with the Reed and Muench method which yields the tissue culture dose for 50% infectivity (TCID_50_) [63]. Briefly, each stock of biological clone was serially diluted in quadruple and then plated with 2 × 10^5 ^CD4 + T cells in a 96-well plate. Virus production was tested in each well with an in-house p24 antigen capture enzyme-linked immunosorbent assay (ELISA).

The growth competition assays were performed in PHA/IL-2 activated PBMC's as described previously [4,8]. Pooled PBMC's from at least four healthy donors were infected with two different viruses at equal multiplicity of infection (0.0005 MOI). Uninfected cultures were used as HIV-1-negative controls and monoinfected cell cultures of each virus provided the positive controls. Virus mixtures were incubated with 2 × 10^5 ^PBMC's at 37°C in 5% CO_2 _for 25 hours, then washed three times with 1 × phosphate buffered saline (PBS) and then resuspended in complete medium [4,7,8,43]. Cell-free supernatant was tested for p24 antigen detection 7 days postinfection with an in-house ELISA [64]. Two aliquots of supernatant and cells were harvested at day 7 after infection and stored at -80°C for further analysis.

### HTA analysis of dual infections

The viral DNA of all dual-infected and mono-infected cultures was extracted from lysed cells with the QIAamp DNA Blood Mini kit (QIAGEN Inc., Valencia Calif.). HIV-1 DNA was amplified by PCR using a set of external primers (ED14: 5'-TCTTGCCTGGAGCTGTTTGATGCCCCAGAC-3' and EnvB: 5'-AGAAAGAGCAGAAGACAGTGGCAATGA-3'), followed by nested primers (E125 5'-CAATTTCTGGGTCCCCTCCTGAGG-3' and E80 5'-CCAATTCCCATACATTATTGTG-3') [65]. Both the external and nested PCRs were carried out in a 100 μl reaction mixture under defined cycling conditions [4,5]. The nested products from *env *(C3 V3) were analyzed with a heteroduplex tracking assay (HTA) to determine the composition of the virus mixture in the competition experiments as described earlier [4,5,7,43]. The radiolabelled DNA probes were amplified from the *env *region using the set of primers described above. For this PCR reaction, one of the primers was labelled with 2 μCi of [γ-32 P] ATP using T4 polynucleotide kinase (PKN, Roche Belgium) [4]. Subsequently, labelled probes were separated on 1% agarose gel and purified with the QIAquick gel extraction kit (QIAGEN Benelux, Venlo, The Netherlands). The HTA reaction mixtures containing DNA annealing buffer (100 mM NaCl, 10 mM Tris-HCl pH 7.8, 2 mM EDTA), 10 μl of amplified DNA from the dual infection/competition culture and 0.1 pmol of radioactive probe were denatured at 95°C for 3 min, incubated at 37°C for 5 min and transferred on wet ice for re-annealing. DNA heteroduplexes were resolved on Criterion 5% TBE non-denaturated polyacrylamide gels (BIORAD Belgium) for 75 min at 200 V. Gels were dried at 80°C for 45 min, exposed and scanned with a phosphor imager (Cyclone, Perkin Elmer Inc., Boston, Mass.) and analyzed with the PerkinElmer OptiQuant software package [5].

### Viral fitness calculations

In the competition experiments, the ratio of two viruses produced in a dual infection was analysed with HTA and compared to the monoinfections [5,65]. The production of individual HIV clones in a dual infection (*f*_*o*_) was divided by the initial proportion in the inoculum (*i*_*o*_). This quotient is referred to as relative fitness (*W *= *f*_*o *_*/i*_*o*_). The ratio of the relative fitness values of each HIV variant in the competition is a measure of the fitness difference (*W*_*D*_) or ratio between two HIV strains (*W*_*D *_= *W*_*M*_*/W*_*L*_), where *W*_*M *_corresponds to the relative fitness of more fit virus and *W*_*L *_corresponds to the relative fitness of less fit virus [4].

### Strain-specific PCR

The results of the competition experiments were confirmed with a virus strain-specific PCR. For this purpose, a specific nested PCR primer set was designed for each virus strain. Primers were located in different parts of *env-V3 *or *gag*; amplified nested PCR products have a distinct length for each strain. The strain specific primer sets were also used for detecting viruses in plasma samples. The amount of DNA was estimated by comparing the intensity of the bands on agarose gels using TINA version 2.09 g.

### Gene expression profile

Early gene expression events after HIV-1 infection of PBMC's were assessed with the RT^2^Profiler™ PCR Array: Human Inflammatory Cytokines and Receptors (SABiosciences, Frederick, MD, USA). Virus stocks were available for the following clones: B1.1, B1.3, B2.5, B3.1, B4.2 and B4.4. For each virus 4.10^5 ^PBMC's, isolated and pooled from 4 different healthy donors, were infected with a MOI of 0.05. Cells were incubated for 2 hours, spun down, and resuspended in RPMI 1640 medium (Invitrogen Corporation, Carlsbad, Calif.) supplemented with L-glutamine, 10% heat-inactivated foetal calf serum and IL-2, and cultured for an additional 4 hours. Total RNA was isolated from the PBMC cultures with the RNeasy^® ^Mini Kit (QIAGEN Benelux B.V., Venlo, The Netherlands). cDNA was synthesized with the RT^2 ^First Strand Kit (SABiosciences, Frederick MD, USA). Real-time PCR reactions were done with the RT2 SYBR Green/ROX qPCR Master Mix (SABiosciences, Frederick, MD, USA), and were analysed with a Taqman 7000 system (ABI, Foster City, CA, USA). Results were compiled with a program available from SABiosciences.

### LTR-constructs and luciferase-assays

The LTR region of the viral genome from the biological clones was amplified by nested PCR, amplifying nt 2-560 of the HXB2 genome [GenBank: K03455]. The outer primer set is located in the *nef *gene and in the U5 region of the LTR. The 5'nested primer contains a *Kpn *I site and is located upstream of the U3 region. Primers were specially designed to amplify LTR's from clones B1-B4. PCR products were digested with *Kpn *I and *Hind *III (a *Hind *III site is present at position 531-536 in the LTR of HIV (HXB2 numbering) and cloned into the pGL3-Basic vector (Promega, Madison, WI). Because a *Kpn *I site is already present in the clone B3.1 LTR sequence, a nested primer with an added *Mlu *I site was used to construct B3.1 LTR-luciferase constructs. Constructs were verified by sequencing.

Human Embryonic Kidney cells 293 T (HEK-293 T cells) were used in all luciferase experiments. Cells were grown at 37°C in Dulbecco's Modified Eagle Medium with 10% Fetal Calf Serum under 5% CO_2 _and transfected using nanofectin (PAA Laboratories GmbH, Pasching, Austria). Mixtures contained 2 ng of different LTR-luciferase constructs (B1, B2, B3, B4 and B(LAI)), 1 ng of pRL-CMV plasmid (Promega, Madison, WI) expressing *Renilla *luciferase as an internal control for transfection efficiency [66], and pBluescript in such a concentration that the total amount of DNA would always be 200 ng. To test the activation of the promoters by tat, constructs were titrated with different concentrations of a tat-expressing plasmid (pTAT). Cells were cultured for two days and lysed in Passive Lysis Buffer (Promega, Madison, WI). Firefly and *Renilla *luciferase activities were determined with the dual-luciferase reporter assay (Promega, Madison, WI) as described previously [66]. The activity of different constructs was calculated as the ratio of the firefly and *Renilla *luciferase activities, and corrected for between-session variation [67].

## Competing interests

The authors declare that they have no competing interests.

## Authors' contributions

MC conceived of the study and designed the experiments. MC and ACK analysed and interpreted the results. ACK drafted the manuscript, and BB critically revised it. KK, KKA, YG and GV performed the competition experiments. FK sequenced the patient materials, did replication experiments and performed the microarray analysis. FK, VRB and KK sequenced the biological clones. SJD cloned the LTR's and performed the luciferase assays. All authors have seen and approved the final version of the manuscript.
